# Activation of AMPK Inhibits Cholera Toxin Stimulated Chloride Secretion in Human and Murine Intestine

**DOI:** 10.1371/journal.pone.0069050

**Published:** 2013-07-30

**Authors:** Ailín C. Rogers, Lisa Huetter, Nadia Hoekstra, Danielle Collins, Anne Collaco, Alan W. Baird, Desmond C. Winter, Nadia Ameen, John P. Geibel, Sascha Kopic

**Affiliations:** 1 University College Dublin and St. Vincent's University Hospital, Dublin, Ireland; 2 Institute of Physiology and Pathophysiology, Paracelsus Medical University, Salzburg, Austria; 3 Department of Surgery, Yale University, School of Medicine, New Haven, Connecticut, United States of America; 4 Department of Pediatrics, Yale University, School of Medicine, New Haven, Connecticut, United States of America; 5 Department of Cellular and Molecular Physiology, Yale University, School of Medicine, New Haven, Connecticut, United States of America; Heidelberg University, Germany

## Abstract

Increased intestinal chloride secretion through chloride channels, such as the cystic fibrosis transmembrane conductance regulator (CFTR), is one of the major molecular mechanisms underlying enterotoxigenic diarrhea. It has been demonstrated in the past that the intracellular energy sensing kinase, the AMP-activated protein kinase (AMPK), can inhibit CFTR opening. We hypothesized that pharmacological activation of AMPK can abrogate the increased chloride flux through CFTR occurring during cholera toxin (CTX) mediated diarrhea.

Chloride efflux was measured in isolated rat colonic crypts using real-time fluorescence imaging. AICAR and metformin were used to activate AMPK in the presence of the secretagogues CTX or forskolin (FSK). In order to substantiate our findings on the whole tissue level, short-circuit current (SCC) was monitored in human and murine colonic mucosa using Ussing chambers. Furthermore, fluid accumulation was measured in excised intestinal loops.

CTX and forskolin (FSK) significantly increased chloride efflux in isolated colonic crypts. The increase in chloride efflux could be offset by using the AMPK activators AICAR and metformin. In human and mouse mucosal sheets, CTX and FSK increased SCC. AICAR and metformin inhibited the secretagogue induced rise in SCC, thereby confirming the findings made in isolated crypts. Moreover, AICAR decreased CTX stimulated fluid accumulation in excised intestinal segments.

The present study suggests that pharmacological activation of AMPK effectively reduces CTX mediated increases in intestinal chloride secretion, which is a key factor for intestinal water accumulation. AMPK activators may therefore represent a supplemental treatment strategy for acute diarrheal illness.

## Introduction

Acute diarrheal illness (ADI) still represents a major health care concern. Children are particularly vulnerable to the lethal effects of ADI: one out of five deaths in children (<5 years) is caused by diarrhea, which is, in theory, preventable [1]. The molecular mechanism underlying many enterotoxin mediated secretory diarrhea entities is an increase in intestinal chloride secretion through apical chloride channels, such as the cystic fibrosis transmembrane conductance regulator (CFTR) [2]. For example, cholera toxin (CTX) exerts its pathophysiological effects by raising the intracellular levels of cAMP in the enterocyte, resulting in protein kinase A (PKA) activation and subsequent CFTR opening and trafficking [2]. This toxin-mediated modulation of physiological intestinal ion transport mechanisms increases luminal osmolarity, which in turn causes fulminant water loss. Past scientific strategies have focused on the development of optimized oral rehydration formulations or small-molecule CFTR inhibitors [3]. In the current report we investigated an alternative scientific approach to inhibit the augmented enterotoxin induced chloride flux by pharmacological modulation of the ubiquitous AMP-activated protein kinase (AMPK).

AMPK is a multi-subunit protein that acts as an intracellular “energy sensor” [4]. In response to cellular stress, such as ischemia or glucose deprivation, it prevents ATP depletion through alteration of metabolic pathways resulting in net energy conservation [4] and is now a target in the treatment of metabolic disorders, such as Diabetes Mellitus type II, and ischemic injury [5]. Undoubtedly, active transport accounts for the majority of energy utilization in epithelia, hence it is not surprising that AMPK has also emerged as a potent modulator of ion transport proteins. For example, we have previously reported that AMPK can serve as an “off-switch” for gastric acid secretion [6], [7]. Of interest for the current investigation are earlier reports demonstrating that AMPK can inhibit chloride flux through CFTR by directly phosphorylating the channel at its regulatory R-domain, thereby decreasing its open probability [8], [9], [10], [11], [12]. Pharmacological activation of AMPK was shown to decrease cAMP stimulated short-circuit current (SCC; an indicator for chloride flux) in cultured monolayers of T84, Calu-3 and MDCK cells [13], [14], [15]. Furthermore, we and other groups have also provided evidence for a regulatory role of AMPK in the process of intestinal ion transport in native tissues [16], [17], [18]. For example, we have demonstrated that hypoxia decreases intestinal baseline chloride secretion, and that inhibition of AMPK can revert the hypoxia induced changes in intestinal ion transport [18].

**Figure 1:**
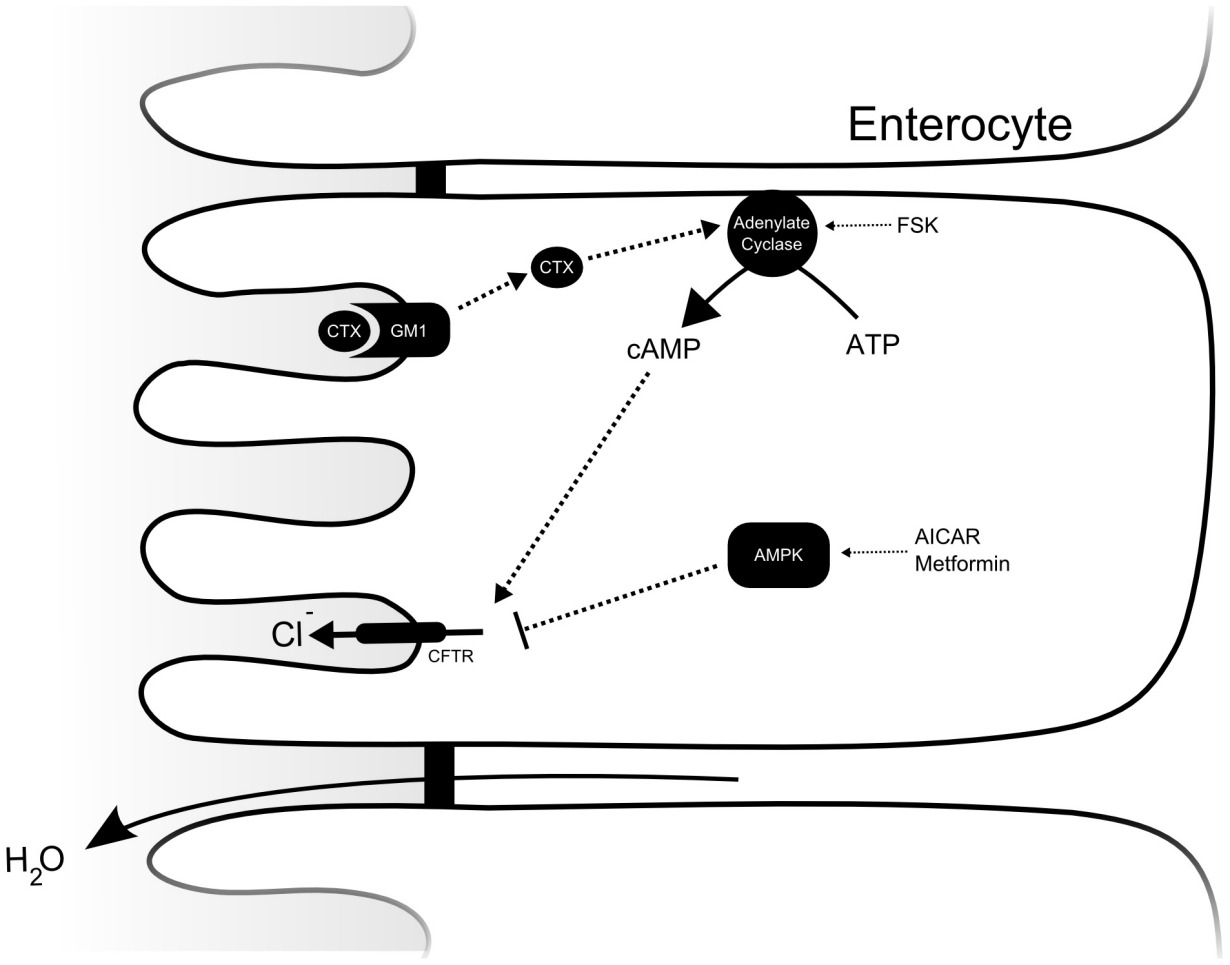
Cell model summarizing the hypothetical inhibitory effect of AMPK activation on CTX induced chloride secretion. CTX binds to the surface ganglioside GM1. Following internalization, the A subunit of the toxin stimulates adenylate cyclase, leading to increased intracellular levels of cAMP and CFTR opening. Secretion of chloride increases luminal osmolarity, resulting in water secretion. Activation of AMPK by AICAR or metformin has an inhibitory effect on CFTR and may therefore abrogate the CTX induced hypersecretion of chloride.

These observations indicate that AMPK functions as a physiological regulator of chloride and concomitant water flux in a broad variety of epithelia, with increased importance in times of physiological stress. In light of this evidence, AMPK emerges as a potential candidate to counteract the deleterious effects of toxin induced secretory diarrhea. We hypothesized that activation of AMPK can abrogate forskolin (FSK) and, more importantly, CTX induced chloride and water flux in the intestine, thereby directly ameliorating the pathophysiological basis of many ADI entities (Figure 1). We have chosen to investigate the underlying hypothesis in a series of assays conducted in murine and human tissue, ranging from single intestinal crypts to epithelial sheets and intact intestinal loops.

## Materials and Methods

### Ethics Approval

The usage of animals and the protocol for isolating intestinal tissue were approved by the Institutional Animal Care and Use Committee at Yale University and are in accordance with the guideline for the proper care and use of animals (IACUC 2012-07654).

For human tissue samples institutional review board approval (St Vincent's University Hospital, Dublin, Ireland) including written informed patient consent was obtained. There were no cases where a parent or guardian was required to give consent. The consent procedure is fully approved by the institutional review board.

### Tissues & Cell Culture

Male Sprague-Dawley rats within a weight range of 250–450 g purchased from Charles River Laboratories. Male C57BL/6 mice were bred in-house. Animals were given free access to water and fed regular chow (PMI Nutrition, Henderson, CO, USA) before investigation.

For Western blot (WB) analysis rats were anaesthetized with thiobutabarbital (Sigma, St. Louis, MO, USA). The abdominal cavity was opened with a longitudinal incision. Jejunal loops were ligated *in situ* and injected with CTX (5 µg/mL; Sigma), or CTX+AICAR (1 mM; Sigma). Sham surgery was performed on control animals. Following injection, the abdominal cavity was closed and the animal was kept under anesthesia. After 4 h the abdominal cavity was reopened, the intestinal loops were extracted for WB analysis and the animal was sacrificed. For Ussing chamber and fluorescent imaging studies animals were anaesthetized and killed by an overdose of isoflurane and tissue was extracted.

Human colon was obtained at surgical resection for colonic carcinoma. The normal histological appearance of tissues was confirmed by routine pathological examination of samples obtained during dissection. Tissues from the resection margins were immediately transferred to the laboratory in preoxygenated Krebs–Henseleit (KH) solution (composition in mM. NaCl 118, KCl 4.7, CaCl_2_ 2.5, MgSO_4_ 1.2, KH_2_PO_4_ 1.2, NaHCO_3_ 25, and glucose 11.1, pH 7.4).

Caco-2_BBE_ (BBE) cells were obtained from the American Type Culture Collection (ATCC) and grown at 37°C in a 5% CO_2_–90% air atmosphere. The culture medium was a high glucose, l-glutamine Dulbecco's Modified Eagle Medium (Gibco, Life Technologies, Grand Island, NY, USA), supplemented with 10% FBS (Gibco), 10 µg/mL apo-transferrin (Sigma), 1 mM Na-pyruvate (Sigma), 1% penicillin-streptomycin (Gibco), 1 µg/mL amphotericin B (Gibco), and 5 µg/mL Plasmocin™ (Invitrogen, Life Technologies, Grand Island, NY, USA). Cells were seeded at a density of 1×10^5^ cells/cm^2^ onto 100-mm cell culture dishes (Corning, Corning, NY, USA) and passed onto 25-mm Transwell® filters (Corning) at 70% confluence. Following establishment of a polarized epithelial layer, CTX (10 µg/mL) was applied apically and 1 mM AICAR was applied bilaterally. Cells were incubated for 4 h at 37°C prior to protein harvesting for WB analysis.

### Isolation of colonic crypts

Colonic crypts were isolated according to a protocol used previously by our laboratory [18], [19]. In brief; segments of rat colon were suspended in a calcium chelation solution (composition in mM: NaCl 96, KCl 1.5, HEPES/Tris 10, NaEDTA 27, Sorbitol 45, Sucrose 28). After 25 min at 37°C in a thermostatically controlled water bath the suspension of isolated crypts was centrifuged at 200 rpm for 1 min. The pellet was resuspended in HEPES-buffered Ringer's solution (composition in mM: NaCl 115, KCL 5, CaCl_2_ 1, MgSO_4_ 1.2, HEPES 32.2, glucose 10; pH 7.4 at 37°C).

### Fluorescence imaging

A suspension of isolated crypts was subsequently applied to a coverslip precoated with the cell adhesive Cell-Tak (BD, Franklin Lakes, NJ, USA). For the duration of the experiment, the coverslip was mounted in a thermostatically controlled chamber (37°C) and superfused with HEPES-buffered Ringer's solution (composition see above). Imaging was conducted on an inverted microscope (Olympus IX71) connected to a digital imaging system (Universal Imaging, Downingtown, PA, USA).

### Measurement of intracellular chloride

For quantification of changes in intracellular chloride, the fluorescent dye N-(ethoxycarbonylmethyl)-6-methocyquinolinium bromide (MQAE; Enzo Life Sciences, Farmingdale, NY, USA) was used. Crypts were loaded with 5 mM MQAE for 30 minutes and then excited at 350±10 nm. The emission signal was measured at 460±10 nm. The intensity of fluorescence was recorded in arbitrary fluorescence units (AFUs). Results are given as changes in AFUs per minute (ΔAFU/sec) after exposing the cells to a chloride free superfusate (composition in mM: NaGluconate 110, KGluconate 5, CaGluconate 2, MgSO_4_ 1.2, HEPES 32.2, glucose 10, pH 7.4 at 37°C). The kinetics of the resulting chloride efflux, measured as changes in intracellular fluorescence/time (ΔAFU/sec), are proportional to the number of open surface chloride channels. Of note, MQAE is a quenching dye. Therefore, an increase in AFUs corresponds to a decrease in intracellular chloride concentrations and an increase in ΔAFU/sec reflects an increase in chloride efflux. The ΔAFU/sec values are suitable markers for chloride efflux if individual conditions are compared relatively to each other. However, they are not suitable as indicators for absolute chloride secretion, due to the lack of dye calibration. Single colonic crypts were incubated with an AMPK activator (AICAR or metformin) for 30 minutes before addition of secretagogue. Crypts were imaged at 5 minutes following addition of FSK or 90 minutes following addition of CTX, corresponding with the peak secretory times of these reagents.

### Immunoblotting

WB analysis was conducted as described previously [20]. Briefly, jejunal mucosa was obtained by gently scraping the longitudinally opened loops with a glass slide. The mucosal scrapings or BBE cells were homogenized in lysis buffer (25 mM HEPES, 10% glycerol, 1% Triton-X-100, pH 7.4) containing protease inhibitors (10 nM iodoactamide, 1 mM phenylmethylsulfonyl fluoride and 2 µg/mL leupeptin). Following centrifugation, supernatant was recovered and protein content was determined using a Coomassie Protein Assay Reagent (Thermo Fisher Scientific, Rockford, IL, USA). Samples were diluted in 2× Laemmli buffer, boiled for 5 min (for CFTR heated at 37°C for 5 min) and loaded on an 8% SDS–polyacrylamide gel. Following protein resolution and electrophoretic transfer onto polyvinylidene fluoride (PVDF) membranes (Bio-Rad, Hercules, CA, USA), membranes were blocked (5% milk in Tween/Tris buffer, pH 7.5) and subsequently incubated with primary antibodies (AME 4991 CFTR, 1∶200; CFTR 217, Cystic Fibrosis Foundation Therapeutics, Bethesda, MD, USA, 1∶500; Phospho-AMPKα Thr172, Cell Signaling, Danvers, MA, USA, 1∶1000) diluted in blocking buffer overnight at 4°C. Secondary antibodies were applied for 1 h at room temperature and protein bands were visualized by chemiluminescence (Thermo Fisher Scientific).

### Ussing chamber experiments

#### Murine tissue

Segments of murine colon were mounted in modified Ussing chambers (Physiologic Instruments, San Diego, CA, USA), bathed in Hepes-buffered saline solution (composition see above) and gassed with 100% O_2_ at 37°C. The transepithelial potential difference (PD) generated by the epithelium was continuously short circuited by passing current across the tissue via agar-salt bridge electrodes (3% agar in 3M KCl) and applying a voltage clamp at 30 second intervals. Short circuit current, SCC (µA/cm^2^), was continuously monitored and recorded by computer. SCC serves as an indicator for intestinal chloride secretion and has been validated previously (4). Tissues were pre-treated with AMPK activators for 30 min or until a stable baseline was obtained. Drugs were added to solutions bathing the basolateral side (except CTX) of the preparation. DMSO concentration in the final solution never exceeded 0.1%

#### Human tissue

Mucosal sheets were stripped of overlying muscle layers by hand dissection and mounted in Ussing chambers (World Precision Instruments, Inc., Stevenage, UK). The tissue was bathed bilaterally in oxygenated (95% O_2_/5% CO_2_) physiological buffer at 37°C. The mucosa was short-circuited as for murine tissue, with the voltage was clamped at 0 mV by a feedback amplifier (World Precision Instruments, Inc., Stevenage, UK) and a MacLab data acquisition system (AD Instruments, Hastings, UK) recording the amount of current required (SCC). Tissues were pre-treated with AMPK activators for 30 min or until a stable baseline was obtained. Drugs were added to solutions bathing the basolateral side (except CTX) of the preparation. DMSO concentration in the final solution never exceeded 0.1%

### Fluid accumulation in isolated jejunal loops

Comparable lengths of mouse jejunal loops were ligated *in situ* and excised. Following excision, loops were placed in a temperature controlled (37°C) and oxygenated bath containing HEPES-buffered Ringer's saline (composition see above). Loops were injected with ∼300 µl of buffer containing CTX (10 µg/ml), CTX+1 mM AICAR or vehicle. The loops were weighed pre-injection, post-injection and after 2 hours. Furthermore, the dry-weight of the loops and the suture weight were recorded. Results are expressed as a ratio of g of secreted fluid per g of dry weight.

### Chemicals and reagents

Forskolin was purchased from MP Biomedicals, LLC. (Solon, OH, USA). CTX (Cholera Toxin from V. cholera, 95% (SDS-PAGE)), AICAR (5-aminoimidazole-4-carboxamide-1-beta-D-riboside-monophosphate), compound C (6-[4-(2-piperidin-1-yl-ethoxy)phenyl]-3-pyridin-4-yl-pyrazolo[1,5-a] pyrimidine) and CFTR Inh-172 were purchased at Sigma (St. Louis, MO, USA). Metformin was obtained from Enzo Life Sciences (Farmingdale, NY, USA).

### Statistical analysis

All data are expressed as means ± SEM. The data was analyzed and compared with an unpaired Student's *t*-test. Statistical significance was assumed at a P<0.05.

## Results

### Isolated colonic crypt measurements

#### AMPK inhibits FSK stimulated chloride secretion in isolated rat colonic crypts

**Figure 2:**
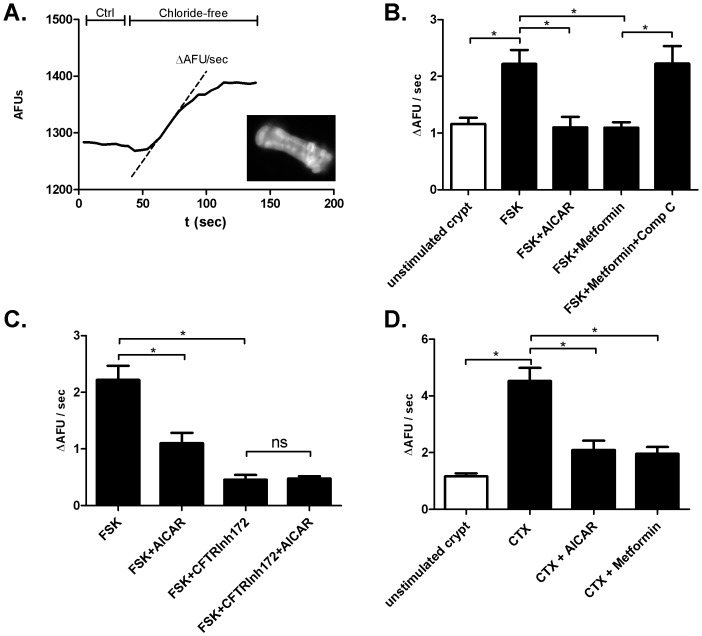
Intracellular chloride measurements in isolated rat colonic crypts. A. Sample tracing of an intracellular chloride measurement using real-time fluorescence microscopy. Switching to a chloride-free superfusate causes chloride efflux and an increase in intracellular fluorescence (quenching dye). The kinetics of the change in the fluorescence signal (ΔAFU/sec) correspond to the number of open chloride efflux pathways. The insert shows an image of an isolated colonic crypt loaded with the chloride indicator dye MQAE. B. Bar graphs summarizing chloride efflux under the individual experimental conditions. Activation of AMPK decreases FSK stimulated chloride efflux from isolated rat colonic crypts. C. CFTR is the main chloride efflux pathway affected by AMPK. D Activation of AMPK decreases CTX stimulated chloride.

Treatment of isolated rat colonic crypts with FSK (5 µM) resulted in an increase in chloride efflux compared to unstimulated controls (control: 1.16 ΔAFU/sec±0.11, n = 7; FSK: 2.22 ΔAFU/sec±0.25 n = 25; p = 0.03). Chloride efflux was significantly reduced when FSK treated crypts were pre-exposed to 4 mM AICAR (FSK+AICAR: 1.10 ΔAFU/sec±0.18, n = 17; p = 0.002) (Figure 2). Treatment of FSK incubated crypts with 4 mM metformin also resulted in a significant decrease in chloride efflux. (FSK+metformin: 1.09 ΔAFU/sec±0.09, n = 9; p = 0.01). Preincubation with the specific AMPK inhibitor compound C (10 µM) completely abolished the effect of metformin on chloride efflux in FSK treated crypts (FSK+metformin+compound C: 2.22 ΔAFU/sec±0.31; n = 11; p = 0.99)(Figure 2).

#### AMPK reduces chloride secretion in isolated rat colonic crypts via inhibition of CFTR

FSK (5 µM) incubated crypts treated with CFTRInh172 (10 µM), a small molecule inhibitor of CFTR, significantly reduced chloride secretion (FSK+CFTRInh172: 0.46 ΔAFU/sec±0.08, n = 17; p<0.0001) The addition of AICAR, however, showed no further decrease in chloride secretion, suggesting that AMPK exerts its inhibitory effects on chloride secretion mostly via inhibition of CFTR. (FSK+CFTRInh172+AICAR: 0.48 ΔAFU/sec±0.04, n = 14; p = 0.84)(Figure 2).

#### AMPK inhibits CTX induced chloride secretion in isolated rat colonic crypts

Isolated crypts of CTX (10 µg/ml) treated colonic segments showed a significant rise in chloride secretion compared to untreated controls. (CTX: 4.52 ΔAFU/sec±0.46, n = 28; p = 0.001). Exposure to AICAR (4 mM) significantly reduced the CTX stimulated chloride efflux (CTX+AICAR: 2.09 ΔAFU/sec±0.32, n = 18; p = 0.0004). Metformin (4 mM) treatment resulted in a comparable reduction in chloride efflux (CTX+metformin: 1.956 ΔAFU/sec±0.24, n = 18; p = 0001) (Figure 2).

### AMPK phosphorylation

**Figure 3:**
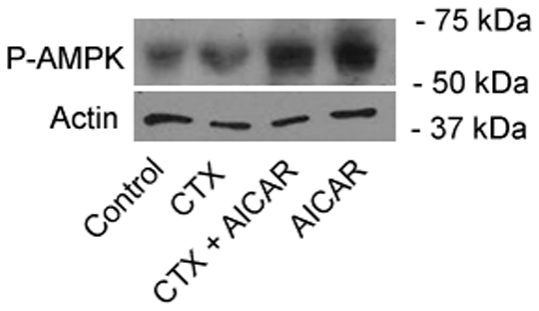
WB analysis of AMPK phosphorylation in BBE monolayers. Phosphorylation of AMPK at Thr172 is a prerequisite for AMPK activation. AICAR treatment resulted in increased phosphorylation of AMPK in polarized BBE monolayers. CTX treatment did not alter the levels of phosphorylated AMPK.

The phosphorylation status of AMPK was investigated in confluent BBE monolayers by WB analysis. These studies were conducted with the aim of assessing whether CTX changes the baseline phosphorylation status of AMPK and to confirm AMPK activation following AICAR (1 mM) treatment (Figure 3). Of note, phosphorylation of AMPK by upstream kinases at Thr172 serves as an indicator for AMPK activation [4]. CTX exposure (10 µg/ml; apical) had no significant effect on the phosphorylation status of AMPK compared to control conditions. AICAR treatment (apical+basolateral) alone and in combination with CTX increased AMPK phosphorylation at Thr172.

### CFTR protein content

**Figure 4:**
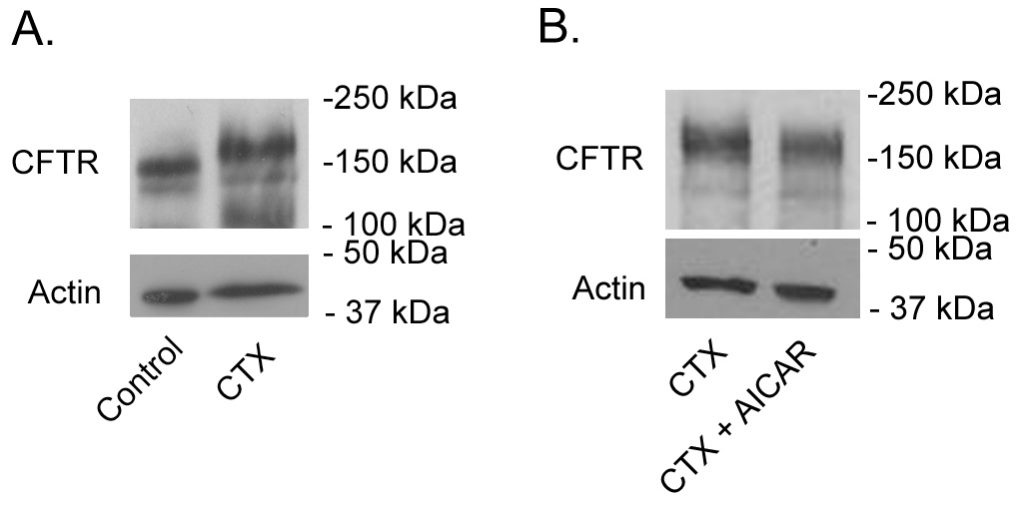
Detection of CFTR by WB analysis of rat jejunum mucosal lysates. Equivalent protein (20 µg) from each condition was analyzed by SDS PAGE. A. CTX does not increase CFTR levels in rat jejunum. B. AICAR treatment does not significantly decrease the total amount of CFTR protein in CTX stimulated jejunum.

WB analysis of jejunal scrapings revealed that CTX treatment (10 µg/ml; luminal) did not increase total levels of CFTR protein compared to untreated control conditions (Figure 4). Similarly, additional AICAR treatment (2 mM; apical) did not appreciably change the total amount of CFTR protein (Figure 4).

### Ussing chamber experiments

#### Murine tissue

**Figure 5:**
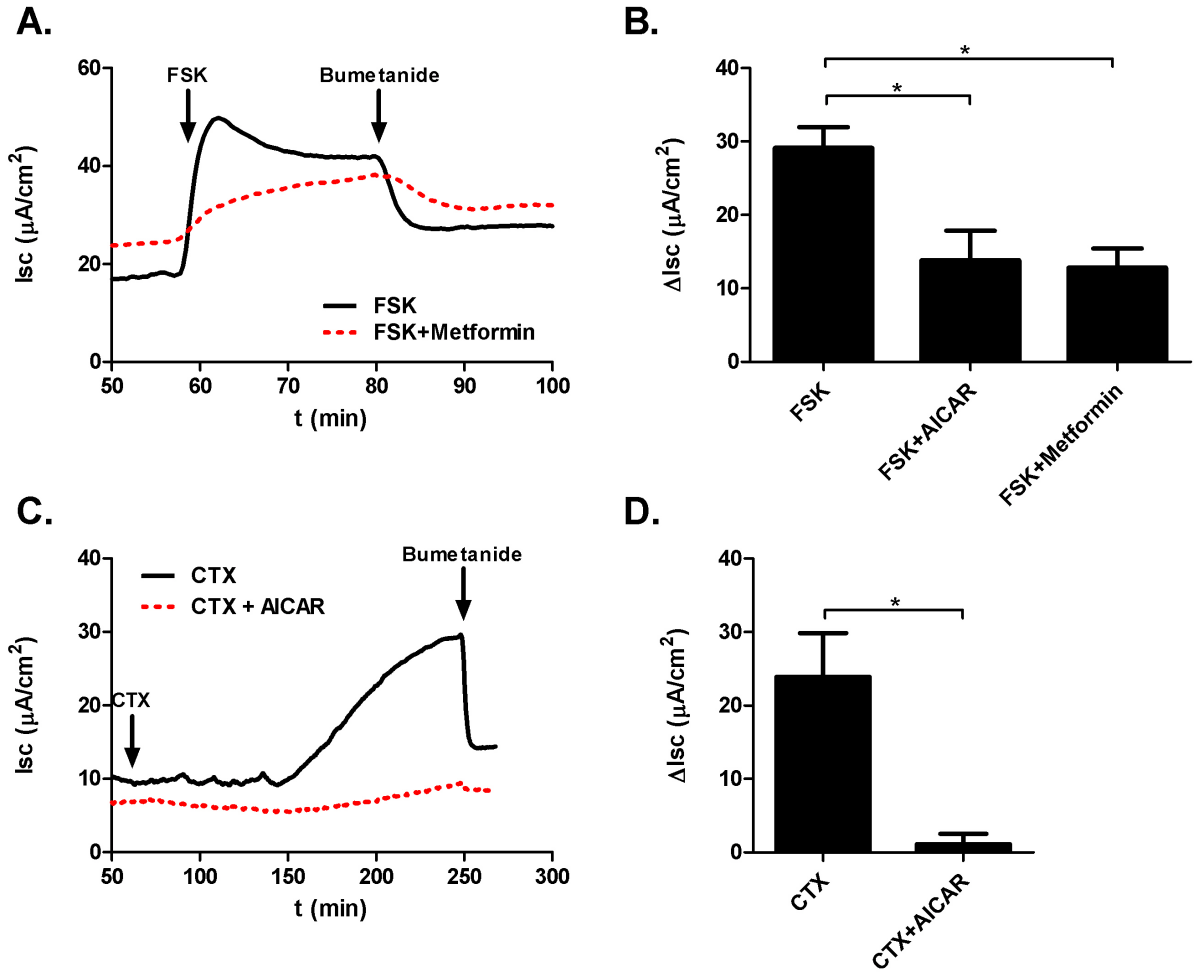
Ussing chamber measurements in mouse colonic mucosal sections. A. Sample tracing demonstrating blunting of FSK stimulated SCC following metformin exposure. SCC is sensitive to bumetanide, indicating that the majority of the current is attributable to chloride secretion B. Bar graphs summarizing changes in SCC following FSK and AMPK activator treatment. C. Sample tracing demonstrating reduction of CTX stimulated SCC after AICAR treatment. D. Bar graphs summarizing changes in SCC following CTX and AICAR treatment.

Addition of FSK (10 µM, basolateral) to the basolateral surface of colonic mucosa resulted in an acute increase in SCC (ΔI_sc_ = 29.11±2.84 µA/cm^2^; n = 14). The FSK-induced rise in SCC was significantly blunted following a 30 min preincubation of the mucoa with AICAR (1 mM, apical+basolateral) (ΔI_sc_ = 13.83±4.02 µA/cm^2^; n = 6; p = 0.008) or metformin (1 mM, apical+basolateral) (ΔI_sc_ = 12.85±2.58 µA/cm^2^; n = 10; p = 0.0005) (Figure 5).

CTX treatment (10 µg/ml, apical) of colonic mucosal sheets increased SCC (ΔI_sc_ = 23.92±5.94 µA/cm^2^; n = 6).AICAR reduced the CTX-mediated rise in SCC (ΔI_sc_ = 1.10±1.40 µA/cm^2^; n = 5; p = 0.007).

#### Human tissue

**Figure 6:**
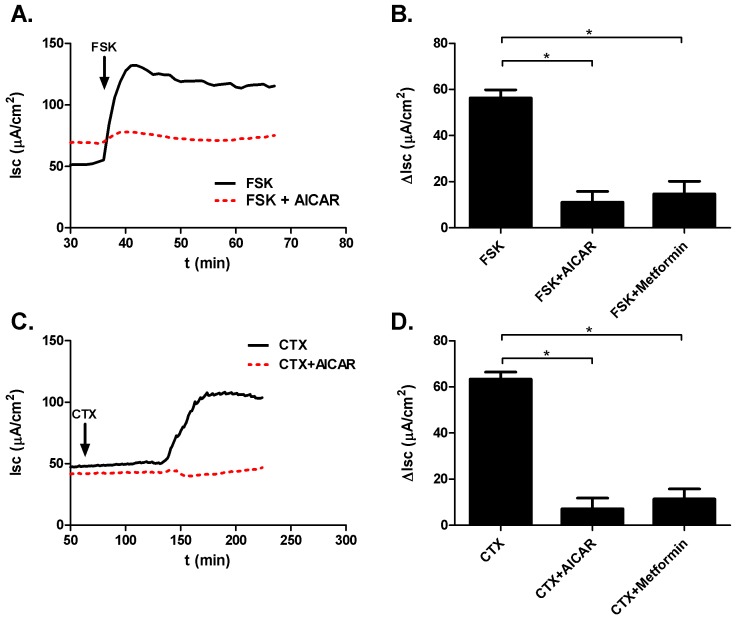
Ussing chamber measurements in human colonic mucosal sections. A. Sample tracing demonstrating blunting of FSK stimulated SCC following AICAR exposure. B. Bar graphs summarizing changes in SCC following FSK and AMPK activator treatment. C. Sample tracing demonstrating reduction of CTX stimulated SCC after AICAR treatment. D. Bar graphs summarizing changes in SCC following CTX and AMPK activator treatment.

In human colonic mucosa, FSK (10 µM) induced a rise in SCC (ΔI_sc_ = 56.37±3.47 µA/cm^2^; n = 6) that was sensitive to treatment with AICAR (3 mM) and metformin (3 mM) (FSK+AICAR: ΔI_sc_ = 11.12±4.65 µA/cm^2^; n = 6; p = 0.0001; FSK+metformin: ΔI_sc_ = 14.70±5.45 µA/cm^2^; n = 6; p = 0.0001), thereby confirming the observations made in murine colonic tissue (Figure 6). Comparable observations were made under CTX (10 µg/ml) stimulation (Figure 6). The CTX induced rise in SCC (ΔI_sc_ = 63.45±3.01 µA/cm^2^; n = 6) was reduced by treatment with the AMPK activators AICAR and metformin (CTX+AICAR: ΔI_sc_ = 7.20±4.54 µA/cm^2^; n = 6; p = 0.0001; CTX+metformin: ΔI_sc_ = 11.45±4.31 µA/cm^2^; n = 6; p = 0.0001).

### Fluid accumulation in isolated intestinal loops

**Figure 7:**
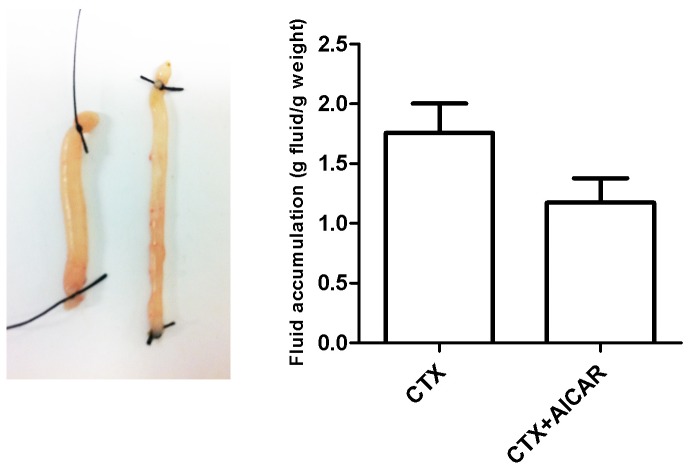
Fluid accumulation in isolated jejunal loops. Left: Note the increased loop swelling of CTX treated loops (left) vs. AICAR+CTX treated loops (right). Right: Quantification of intestinal fluid accumulation.

Following CTX (10 µg/ml) treatment average jejunal loop fluid accumulation amounted to 1.76±0.24 g secreted fluid/g dry weight (n = 10). AICAR significantly decreased the fluid accumulation to 1.16±0.20 g secreted fluid/g dry weight (n = 10; p = 0.006) (Figure 7).

## Discussion

This study examined the effects of AMPK activation on cAMP stimulated intestinal ion secretion. In its role as cellular energy conserver, AMPK modulates epithelial ion transport mechanisms, including CFTR [8], [9], [10], [13], [14], [16], [17]. For example, we have previously presented evidence for AMPK mediated modulation of intestinal chloride transport during states of hypoxia [18]. Enhanced chloride efflux through intestinal CFTR also creates the osmotic driving force underlying intestinal water accumulation in choleraic diarrhea. The pivotal role of CFTR in the pathophysiology of cholera is corroborated by the observation that CFTR deficient animals are immune to the effects of CTX [21]. Given the inhibitory role of AMPK on CFTR, we hypothesized that AMPK can be utilized as a pharmacological target to negate the hypersecretion of chloride that occurs not only in the course of cholera, but also during many other enterotoxigenic diarrhea entities, such as enterotoxigenic *E.coli* mediated diarrhea (Figure 1). We present evidence suggesting that pharmacological activation of AMPK indeed ameliorates the intestinal ion transport response to CTX in a secretory diarrhea model (Figures 2,5,6). To test our hypothesis, we employed assays that allowed us to measure chloride or water secretion in a tiered approach, ranging from single intestinal crypts, to tissue sections and whole excised intestinal loops. Furthermore, key experiments were repeated in human tissue.

The isolated crypt model allows measurement of chloride flux in an intact physiological unit in real-time [18]. The fluorescence measurements demonstrated that chloride efflux from an isolated colonic crypt can be stimulated by FSK (Figure 2). FSK causes accumulation of intracellular cAMP, by activation of the adenylate cyclase, thereby emulating the intracellular effects of CTX. The secretion of fluid is governed by chloride efflux. For example, isolated microperfused colonic crypts were shown to secrete fluid following elevation of intracellular cAMP levels [22]. Activation of AMPK by either AICAR or metformin significantly decreased the amount of chloride efflux, and therefore most likely also water secretion (Figure 2). To substantiate our findings in a pathophysiological context, isolated colonic crypts were treated with CTX. CTX treatment resulted in an augmented chloride efflux, which was sensitive to AMPK activation by either AICAR or metformin (Figure 2). We therefore concluded that AMPK can significantly reduce CTX stimulated chloride efflux at the level of the single intestinal crypt and directly on epithelial cells, thereby abrogating the molecular mechanism preceding intestinal water accumulation.

It has been proposed that some of the effects of metformin are not specific to activation of AMPK. For example, Kongsuphol et al. have demonstrated that phenformin (a drug closely related to metformin) decreased SCC in colonic tissue of AMPK α1 (−/−) mice [17]. In our isolated colonic crypt model, however, the inhibitory effects of metformin on chloride efflux were reversed by simultaneous incubation with the specific AMPK inhibitor compound C, suggesting that metformin indeed exerts its effects via AMPK (Figure 2). Although metformin indisputably also targets other intracellular mechanisms, it was included in our experimental protocols. Metformin is in use by millions of diabetes type II patients, which gives testimony to its safe usage profile. In light of its clinical establishment and its effects on intestinal ion transport, metformin may represent a viable treatment option for ADI. In analogy to previously published reports by other groups, the concentrations of AMPK activators used in our models are rather high (low millimolar range), which may necessitate a future local intestinal delivery mechanism or the development of a specific small-molecule AMPK activator to achieve optimal clinical results [13], [14], [15].

We further attempted to identify the molecular basis of the chloride efflux. In general, it is well accepted that CFTR plays a major role in the process of apical chloride secretion in the intestine. The experiments employing the specific small-molecule CFTR inhibitor (CFTRInh172) suggest that the main mediator of chloride efflux in our system is CFTR (Figure 2). Of note, other apical chloride channels, such as calcium-activated chloride channels or CLC-2 are known to be expressed in the colonic mucosa and may therefore be modulated by AMPK. However, the observation that activation of AMPK in the presence of the CFTR inhibitor does not lead to a further decrease in chloride efflux supports our hypothesis that AMPK mostly modulates CFTR mediated chloride movement [23]. Given the central role of CFTR in the context of secretory diarrhea, other groups have effectively demonstrated that the CFTRInh172 has potent anti-secretory effects in native murine tissues [24]. Although CFTRInh172 effectively ameliorates CTX mediated secretion in experimental models, it is as of now unavailable in a clinical environment [24]. Activation of AMPK via utilization of already clinically available substances, such as metformin, may therefore represent a relevant treatment approach for secretory diarrhea.

It has been shown in other tissues that AMPK can regulate the amount of surface and total ion channels, such as the epithelial sodium channel (ENaC), by initiating their degradation via the ubiquitination pathway [25]. With regard to CFTR the current paradigm suggests that AMPK exerts its inhibitory effects by modulating channel gating through phosphorylation of the channel's R-domain [9], [10], [11]. To gain a deeper understanding of the mechanism underlying AMPK mediated CFTR inhibition a series of WB experiments was conducted in native rat jejunal tissue. WB analysis revealed that 4 h of CTX treatment does not increase total CFTR protein levels (Figure 4). Furthermore, concomitant AICAR treatment did not appreciably alter CFTR levels compared to CTX treatment alone. Although our findings do not allow conclusions on CFTR trafficking – with the total amount of CFTR protein remaining constant – they further substantiate the current working model, which postulates that AMPK changes channel gating rather than channel numbers.

AMPK phosphorylation at Thr172 is a prerequisite for AMPK activation. Interestingly, CTX did not result in AMPK phosphorylation at Thr172 (Figure 3). This is of significance with regard to a potential therapeutic application of AMPK activators in the context of secretory diarrhea, as baseline unphosphorylated AMPK during cholera allows for full pharmacological leverage.

We have analyzed the response of whole colonic mucosal sections to CTX, FSK and AMPK activators with the aim of testing our hypothesis on the whole-tissue level. Although SCC is the product of all ionic currents across the epithelium, it is commonly used as an indicator for chloride flux in intestinal tissue [26]. Both FSK and CTX stimulated responses in SCC were significantly blunted by treatment with AMPK activators (Figures 5,6). We therefore conclude that whole tissue portions behave comparably to isolated single crypts in terms of chloride secretion and that AMPK activation is an effective tool for reversing the CTX induced changes in SCC. Importantly, the effects of AMPK activation were reproducible in human colonic mucosa, indicating that the murine model is relevant to human biology (Figure 6).

Although the hypersecretion of chloride is a key component of the mucosal response to CTX exposure, it should be noted that CTX is known to exert effects beyond CFTR activation. For example, it has been shown that enterotoxigenic changes in luminal osmolarity (the driving force for water accumulation) are mostly a product of (i) augmented secretion of chloride, but also of (ii) decreased absorption of sodium [27]. The apical Na,H-exchanger (NHE) is a key mediator of electroneutral salt absorption and is known to be inversely regulated to CFTR; i.e. increases in cAMP levels inhibit NHE and stimulate CFTR. To date there are no reported effects of AMPK on the activity of the apical NHE isoforms 2 or 3. However, activation of AMPK has been shown to stimulate basolateral NHE1 in human embryonic kidney (HEK) cells [28]. It is therefore plausible that AMPK is also capable of modulating apical NHE isoforms, albeit, as of now this hypothesis is of speculative nature.

In summary, we propose that activation of AMPK is an effective way of ameliorating the intestinal hypersecretion of chloride, which is a molecular key mechanism underlying secretory diarrhea. Our *ex vivo* results supply evidence for a potentially therapeutic effect of AMPK activation on intestinal ion handling during ADI. Ultimately *in vivo* animal experiments remain to be conducted to substantiate this promising outlook.
